# The advantages of endophyte-infected over uninfected tall fescue in the growth and pathogen resistance are counteracted by elevated CO_2_

**DOI:** 10.1038/s41598-017-07183-y

**Published:** 2017-07-31

**Authors:** Wei Chen, Hui Liu, Wurihan, Yubao Gao, Stuart D. Card, Anzhi Ren

**Affiliations:** 1 College of Life Sciences, Nankai University, Tianjin, China; 2 AgResearch Ltd, Grasslands Research Centre, Palmerston North, New Zealand

## Abstract

Atmospheric CO_2_ concentrations are predicted to double within the next century. Despite this trend, the extent and mechanisms through which elevated CO_2_ affects grass-endophyte symbionts remain uncertain. In the present study, the growth, chemical composition and pathogen resistance of endophyte-infected (E+) and uninfected (E−) tall fescue were compared under elevated CO_2_ conditions. The results showed that the effect of endophyte infection on the growth of tall fescue was significantly affected by elevated CO_2_. Significant advantage of E+ over E− tall fescue in tiller number, maximum net photosynthetic rate and shoot biomass occurred only under ambient CO_2_. With CO_2_ concentration elevated, the beneficial effect of endophyte infection on the growth disappeared. Similarly, endophyte infection reduced lesion number and spore concentration of *Curvularia lunata* only under ambient CO_2_. These results suggest that the beneficial effect of endophyte infection on the growth and pathogen resistance of tall fescue could be counteracted by elevated CO_2._ An explanation for the counteraction may be found in a change in photosynthesis and nutritive quality of leaf tissue.

## Introduction

Atmospheric carbon dioxide (CO_2_) is expected to rise from a current ambient concentration of 390 ppm to between 550 and 1000 ppm by the year 2100^1^. CO_2_ enrichment in the atmosphere in general stimulates photosynthetic activity and growth of C_3_ plants^2^. CO_2_ enrichment has also been shown to change plant resource allocation, especially plant C:N ratio^3^. The carbon-nutrient balance (CNB) hypothesis predicts that carbon products in excess of those needed for primary metabolic functions will result in increased carbon-based secondary metabolites and subsequent decreased N-based secondary metabolites^4^. Such alterations in plant primary and secondary metabolism are expected to alter the availability of photosynthates and defensive compounds for plant-associated microbes^5^, modifying plant-microbial interactions such as plant-rhizobial symbiosis^6^, mycorrhizae^7^, and plant-endophyte complexes^8, 9^.

Endophytic fungi within asymptomatic aerial tissues of plants represent a ubiquitous component of terrestrial plant communities^10^. Among them, the symbiosis between cool season grasses and *Epichloë* endophytes is most common and considered to have important ecological implications^11^. In the symbiosis, the host grasses provide photosynthates and nutrients to the endophytes^12^, and in turn, the endophytes can benefit grasses through increased growth^13, 14^, and by providing tolerance to abiotic and biotic stresses^11, 15–20^. Increasing evidence shows that the endophyte-host interaction depends in many cases, on resource availability^9^. In fact, many of the studies that have found improved growth and resistance in endophyte-infected grasses were done under benign conditions of moderate to high soil nutrient availability^15, 21–23^.

Many studies have shown that elevated CO_2_ increases mycorrhizal colonization of roots and alters plant – mycorrizae interactions^24–27^. Similar to mycorrhiza, fungal endophytes depend on carbon and energy provided by their host plants. However, up to now, studies examining responses of grass-endophyte associations to elevated CO_2_ are limited^8, 9, 28–30^. In the pioneering study, Marks and Clay^29^ found in perennial ryegrass (*Lolium perenne*, a C_3_ plant) and purpletop grass (*Tridens flavus*, a C_4_ plant), the growth of endophyte-infected (E+) and uninfected (E−) plants responded similarly to CO_2_ enrichment. Also in perennial ryegrass, Hunt, *et al*.^8^ reported that E+ biomass tended to be greater than E− plants only at elevated CO_2_, and they further found that E− plants had 40% lower concentrations of soluble protein under elevated CO_2_ than under ambient CO_2_, but this CO_2_ effect on soluble protein was absent in E+ plants. In tall fescue (*Lolium arundinaceum*), Newman, *et al*.^9^ did not find interaction between CO_2_ concentration and endophyte infection in the growth, but they found soluble crude protein concentration increased under elevated CO_2_ for E− plants but not for E+ plants. Ryan, *et al*.^30^ reported that endophyte-derived alkaloids decreased in response to elevated CO_2_. Taken together, the effects of endophyte infection on herbage quality as well as defensive chemistry can be affected by elevated CO_2_. Therefore, the endophyte-induced herbivore^11^ and pathogen resistance^18, 31^ of the host are likely to be impacted by elevated CO_2_ in the atmosphere.

Recently, the effect of *Epichloë* endophyte infection on pathogen resistance has been extensively investigated. The pioneering research by Shimanuki and Sato^32^ demonstrated that timothy plants (*Phleum pratense*) infected by *Epichloë typhina* were resistant to the fungus *Cladosporium phlei*. In *in vitro* investigations, White and Cole^33^, Siegel and Latch^34^ and Christensen^35^ found that *Epichloë* isolates inhibited the growth of pathogenic fungi, only the antifungal activity of endophytes differed between the isolates. In *in planta* investigations, the positive effect of endophyte infection on pathogen resistance of the host grass has been observed in tall fescue^36, 37^, ryegrass^38–41^ and other native grasses^42–44^. Certainly, endophytes do not always improve disease resistance of the host. Negative^45, 46^ and neutral^47, 48^ effects have also been reported. In our previous study^44^, we found that endophyte could enhance pathogen resistance of *Leymus chinensis*, and this endophytic benefit was strengthened by drought treatment. These different reports suggest that the interactions between endophytes and pathogens are complex, and may be affected by species difference as well as environmental factors^31^ such as elevated CO_2_ concentration in the atmosphere.

In the present study, E+ and E− tall fescue were planted under contrasting CO_2_ availability regimes to test the effect of the endophyte infection and CO_2_ concentration on the performance in terms of growth, chemical composition and pathogen resistance of tall fescue. Specifically, we addressed the following questions: (1) does endophyte infection improve growth and pathogen resistance of the grass host? (2) does elevated CO_2_ affect growth and pathogen resistance pattern of tall fescue – endophyte associate? If this is the case, (3) what is the mechanism involved might be?

## Results

### Plant height, leaf number and tiller number

**Figure 1:**
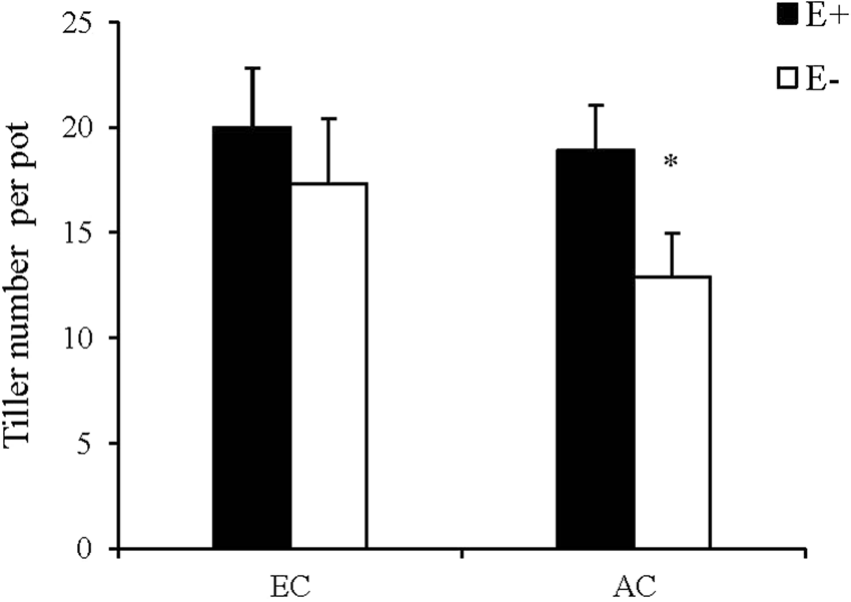
Comparison of tiller number of endophyte-infected (E+) or uninfected (E−) *Festuca arundinacea* under elevated CO_2_ (EC) and ambient CO_2_ (AC) conditions. *Meant significant difference at 0.05 level.

Plant height was only significantly affected by N availability (Table 1), and N supply increased plant height (HN = 54.19 ± 4.80; LN = 31.61 ± 2.15; cm). Leaf number was significantly increased by elevated CO_2_ concentration, N supply as well as endophyte infection (EC = 72 ± 28.6, AC = 63 ± 22.4; HN = 90 ± 15.3; LN = 45 ± 8.3; E+ = 75 ± 25.1, E− = 60 ± 24.7). Tiller number was significantly affected by interaction between CO_2_ concentration and endophyte infection (Table 1, Fig. 1). Under ambient CO_2_ condition, tiller number of E+ was significantly more than that of E−, but under elevated CO_2_ condition, no significant difference occurred.

**Table 1:** Three-way ANOVA for growth characters of endophyte-infected (E+) or uninfected (E−) *Festuca arundinacea* under various CO_2_ and nitrogen conditions.

	Plant height		Tiller number		Leaf number		Maximum net photosynthetic rate		Shoot biomass		Root biomass		Root:Shoot ratio		Leaf carbon concentration		Leaf nitrogen concentration		C:N ratio	
	*F*	*P*	*F*	*P*	*F*	*P*	*F*	*P*	*F*	*P*	*F*	*P*	*F*	*P*	*F*	*P*	*F*	*P*	*F*	*P*
Endophyte (E)	0.10	0.76	39.94	**0**.**00**	33.76	**0**.**00**	3.25	0.08	24.27	**0**.**00**	2.59	0.12	0.16	0.69	4.22	**0**.**05**	12.84	**0**.**00**	11.97	**0**.**00**
CO_2_ (C)	0.96	0.34	15.96	**0**.**00**	11.19	**0**.**00**	347.42	**0**.**00**	433.83	**0**.**00**	11.03	**0**.**00**	2.03	0.16	0.13	0.72	148.34	**0**.**00**	177.49	**0**.**00**
Nitrogen (N)	329.10	**0**.**00**	453.03	**0**.**00**	307.93	**0**.**00**	276.61	**0**.**00**	4722.89	**0**.**00**	216.40	**0**.**00**	0.50	0.48	143.99	**0**.**00**	143.33	**0**.**00**	117.76	**0**.**00**
E × C	0.50	0.48	5.75	**0**.**02**	1.52	0.23	6.65	**0**.**02**	13.55	**0**.**00**	0.16	0.69	0.21	0.65	0.14	0.71	3.27	0.08	0.21	0.65
E × N	0.04	0.85	0.05	0.83	0.28	0.60	13.16	**0**.**00**	8.72	**0**.**01**	2.38	0.13	0.90	0.35	1.71	0.20	0.67	0.42	2.99	0.09
C × N	0.31	0.58	14.82	**0**.**00**	9.43	**0**.**00**	0.03	0.87	269.27	**0**.**00**	8.33	**0**.**01**	0.03	0.86	0.51	0.48	33.33	**0**.**00**	0.01	0.91
E × C × N	0.11	0.75	1.19	0.28	0.52	0.47	4.12	**0**.**05**	7.65	**0**.**01**	0.84	0.37	1.58	0.22	0.94	0.34	4.74	**0**.**04**	2.65	0.11

Significant *P*-values are in bold.

### Maximum net photosynthetic rate and biomass

**Figure 2:**
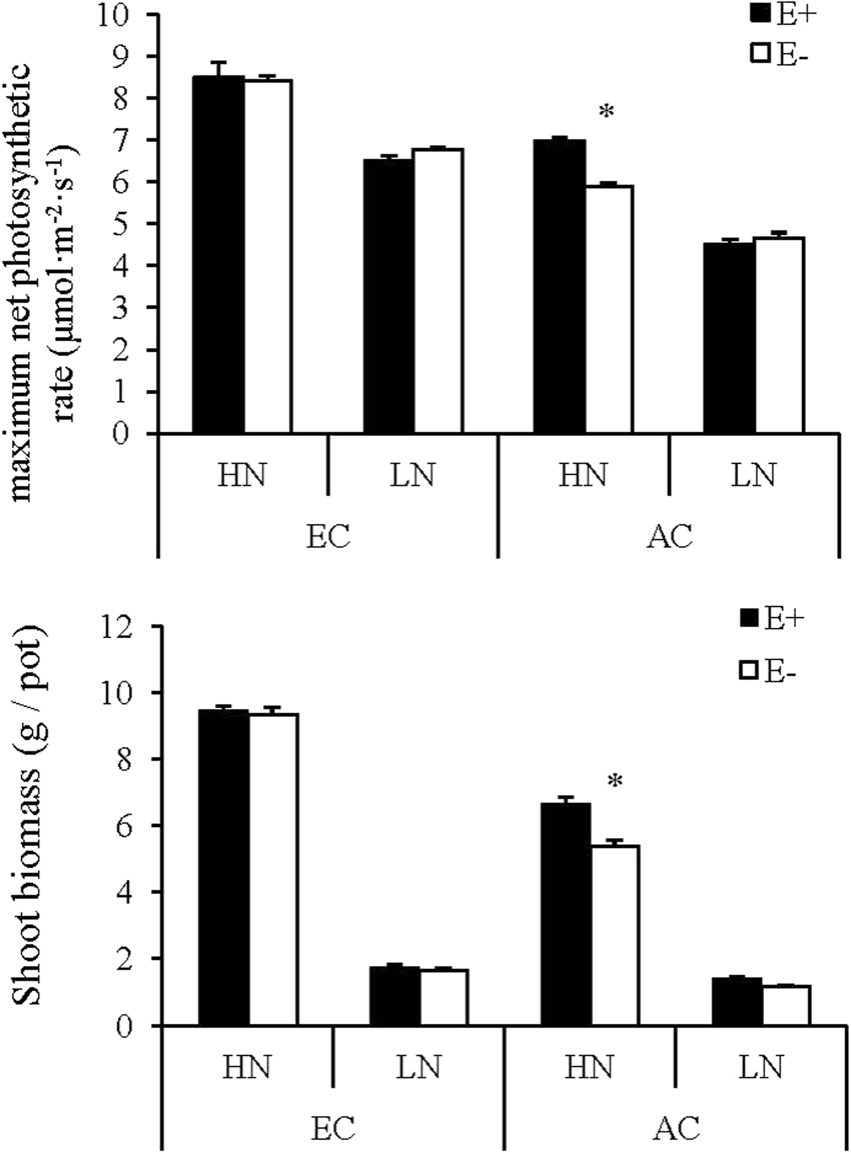
Comparison of maximum photosynthetic rate and shoot biomass of endophyte-infected (E+) or uninfected (E−) *Festuca arundinacea* under different CO_2_ and nitrogen levels. *Meant significant difference at 0.05 level.

Maximum net photosynthetic rate and shoot biomass were significantly affected by three-way interaction among CO_2_ concentration, N supply and endophyte infection (Table 1). Only under ambient CO_2_ and high N condition, both maximum net photosynthetic rate and shoot biomass were greater in E+ than in E− plants (Fig. 2).

### Leaf carbon, nitrogen and C:N ratio

**Figure 3:**
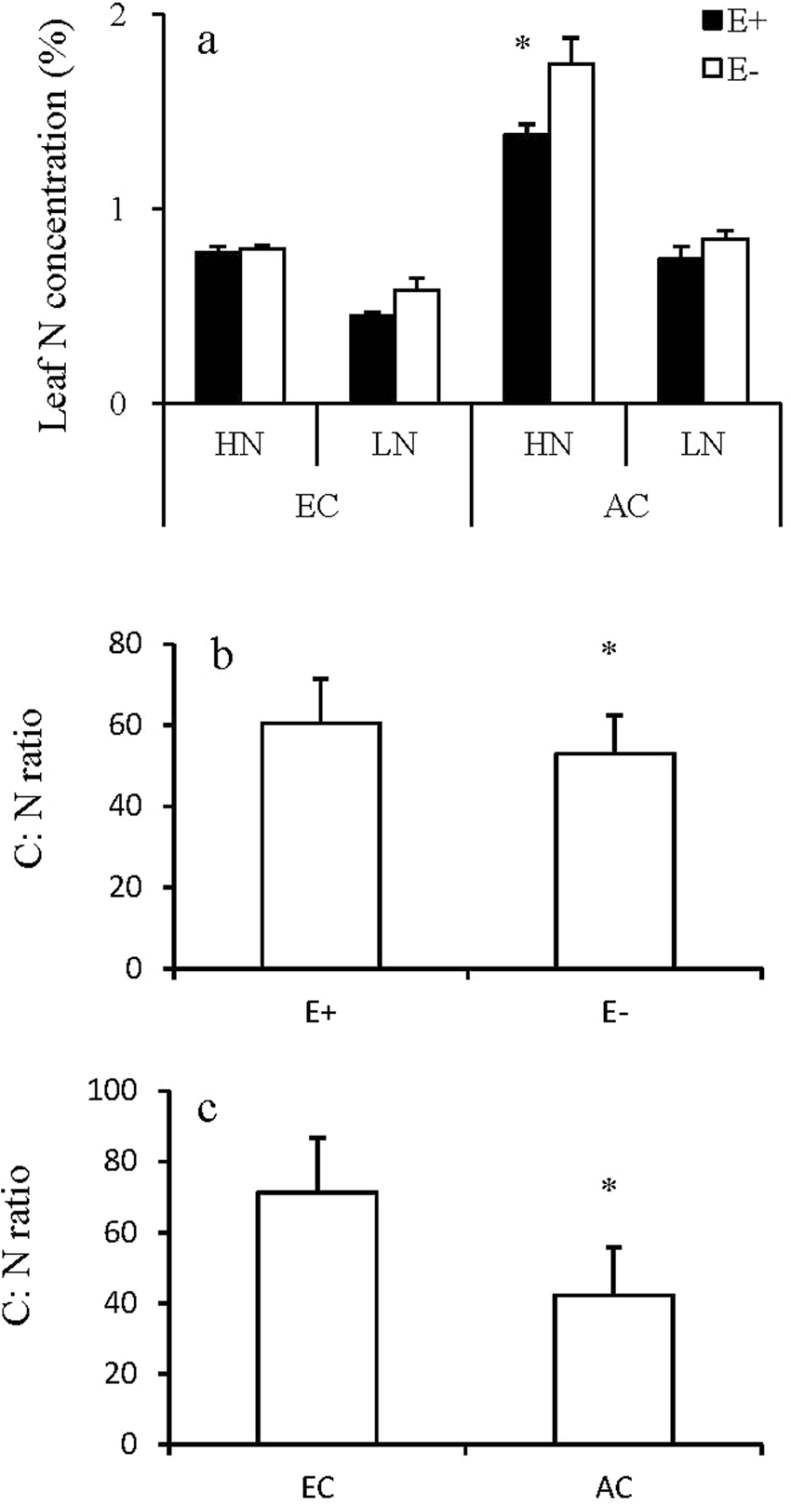
Leaf N concentration of endophyte-infected (E+) or uninfected (E−) *Festuca arundinacea* under different CO_2_ and nitrogen levels (**a**). Leaf C:N ratio of *Festuca arundinacea* under different endophyte (**b**) and CO_2_ treatments (**c**). *Meant significant difference at 0.05 level.

Leaf C concentration was significantly affected by N supply and endophyte infection (Table 1). Leaf N concentration was significantly affected by three-way interaction among CO_2_ concentration, N supply and endophyte infection. Leaf N concentration of E+ plants was lower than that of E− plants only under ambient CO_2_ and high N condition (Fig. 3a). Both elevated CO_2_ concentration and endophyte infection significantly improved leaf C:N ratio (Fig. 3b,c).

### Lesion number and spore concentration of the pathogen

**Figure 4:**
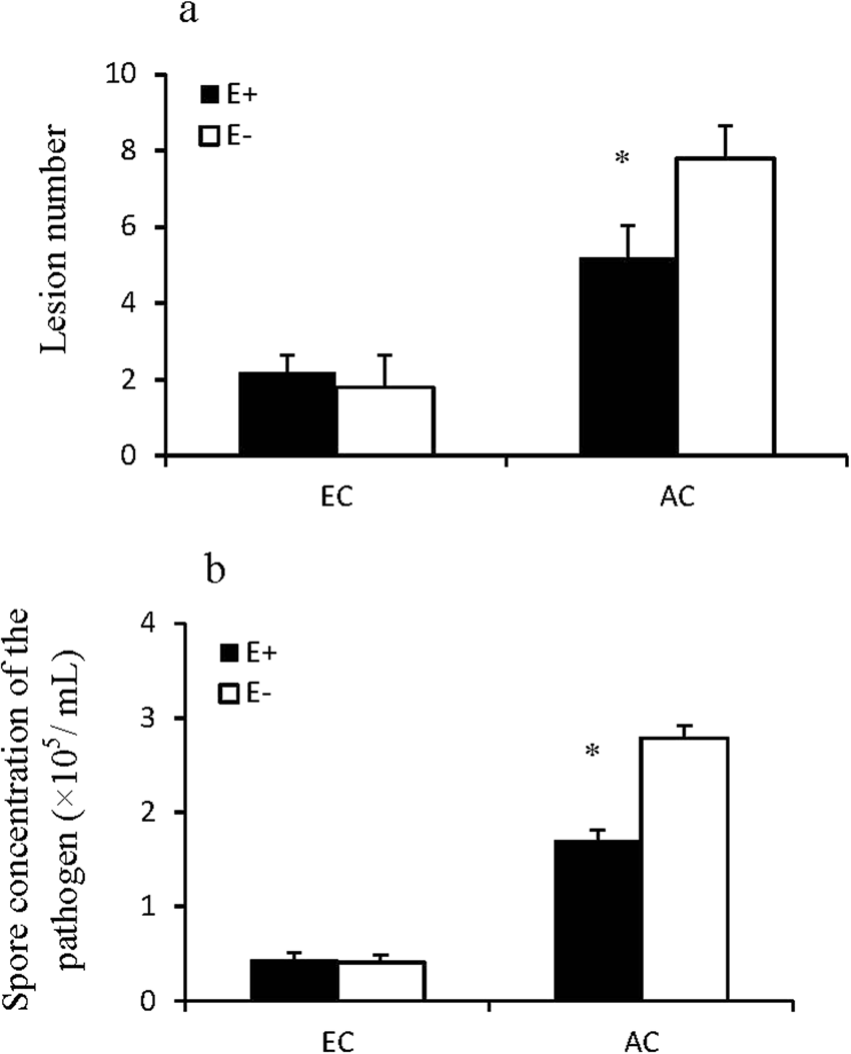
Lesion number (**a**) and pathogen spore concentration (**b**) of endophyte-infected (E+) or uninfected (E−) *Festuca arundinacea* under elevated CO_2_ (EC) and ambient CO_2_ (AC) treatments.*Meant significant difference at 0.05 level.

Both lesion number and pathogen spore concentration were significantly affected by the interaction between CO_2_ concentration and endophyte infection (Table 2). Under ambient CO_2_ concentration, endophyte infection reduced lesion number and pathogen spore concentration of the host leaves when exposed to *Curvularia lunata*. Elevated CO_2_ significantly improved pathogen resistance of both E+ and E− plants. However, no difference occurred in either lesion number or pathogen spore concentration between E+ and E− plants under elevated CO_2_ (Fig. 4). That is to say, the advantage in pathogen resistance of E+ over E− plants was alleviated by elevated CO_2_.

**Table 2:** Three-way ANOVA for pathogen and physiological indices of endophyte-infected (E+) or uninfected (E−) *Festuca arundinacea* under various CO_2_ and pathogen conditions.

	Lesion number		Spore concentration		Soluble sugar concentration		Lignin concentration		RF1		RF2		RF3		RF4	
	*F*	*P*	*F*	*P*	*F*	*P*	*F*	*P*	*F*	*P*	*F*	*P*	*F*	*P*	*F*	*P*
Endophyte (E)	10.52	**0**.**01**	137.45	**0**.**00**	13.60	**0**.**00**	1.47	0.23	0.00	0.99	1.23	0.28	0.81	0.38	4.19	**0**.**05**
CO_2_ (C)	176.09	**0**.**00**	1662.69	**0**.**00**	16.44	**0**.**00**	14.40	**0**.**00**	12.07	**0**.**00**	28.94	**0**.**00**	0.93	0.34	0.00	0.96
Pathogen (P)	—	—	—	—	1.03	0.32	282.45	**0**.**00**	0.17	0.68	0.61	0.44	2.08	0.16	1.03	0.32
E × C	19.57	**0**.**00**	157.03	**0**.**00**	0.09	0.76	1.79	0.19	0.11	0.75	0.11	0.74	0.23	0.64	3.57	0.07
E × P	—	—	—	—	0.01	0.93	0.88	0.36	0.03	0.88	0.09	0.77	0.26	0.61	0.39	0.54
C × P	—	—	—	—	0.00	0.98	14.30	**0**.**00**	6.04	**0**.**02**	12.10	**0**.**00**	8.35	**0**.**01**	0.00	0.98
E × C × P	—	—	—	—	0.45	0.51	3.91	**0**.**05**	0.02	0.89	0.05	0.83	0.69	0.41	0.56	0.46

Signiflicant *P*-values are in bold.

### Soluble sugar and amino acids

**Figure 5:**
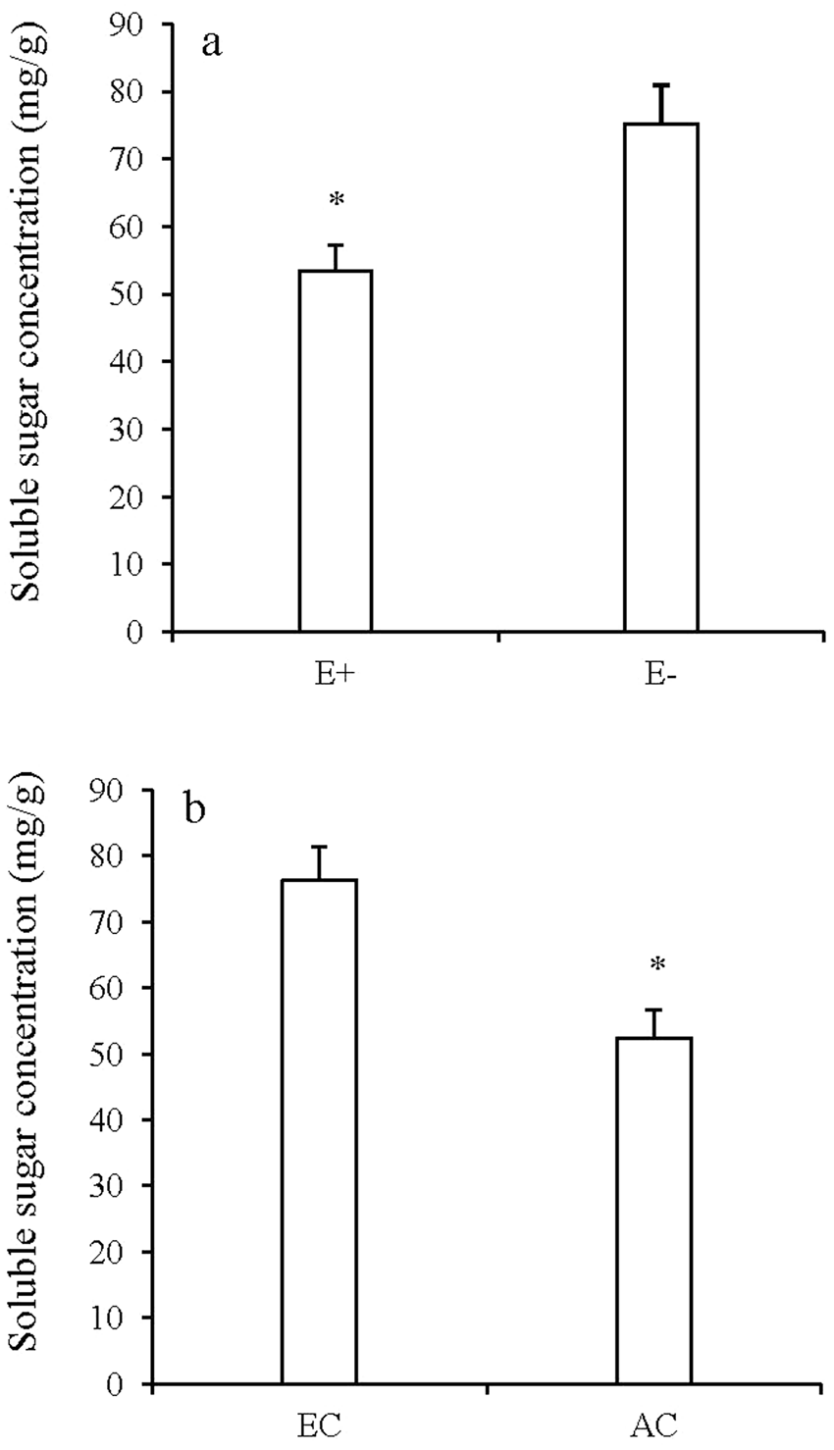
Soluble sugar concentration of *Festuca arundinacea* under different endophyte (**a**) and CO_2_ treatments (**b**). *Meant significant difference at 0.05 level.

Soluble sugar concentration was significantly affected by CO_2_ concentration and endophyte infection (Table 2). Elevated CO_2_ significantly increased soluble sugar concentration while endophyte infection significantly decreased soluble sugar concentration of tall fescue (Fig. 5).

**Figure 6:**
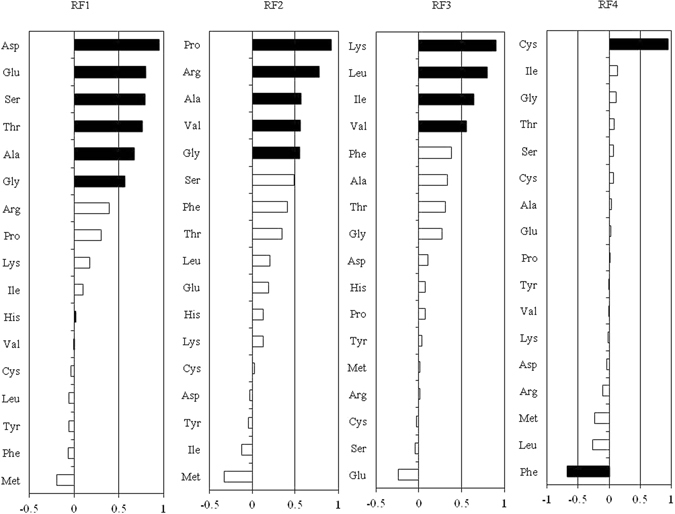
Loadings for each individual amino acid of *Festuca arundinacea* onto the first four rotated factors (RF). The individual amino acids loading heavily either positively (loading ≥ +0.5) or negatively (loading ≤ −0.5) are highlighted in black.

**Figure 7:**
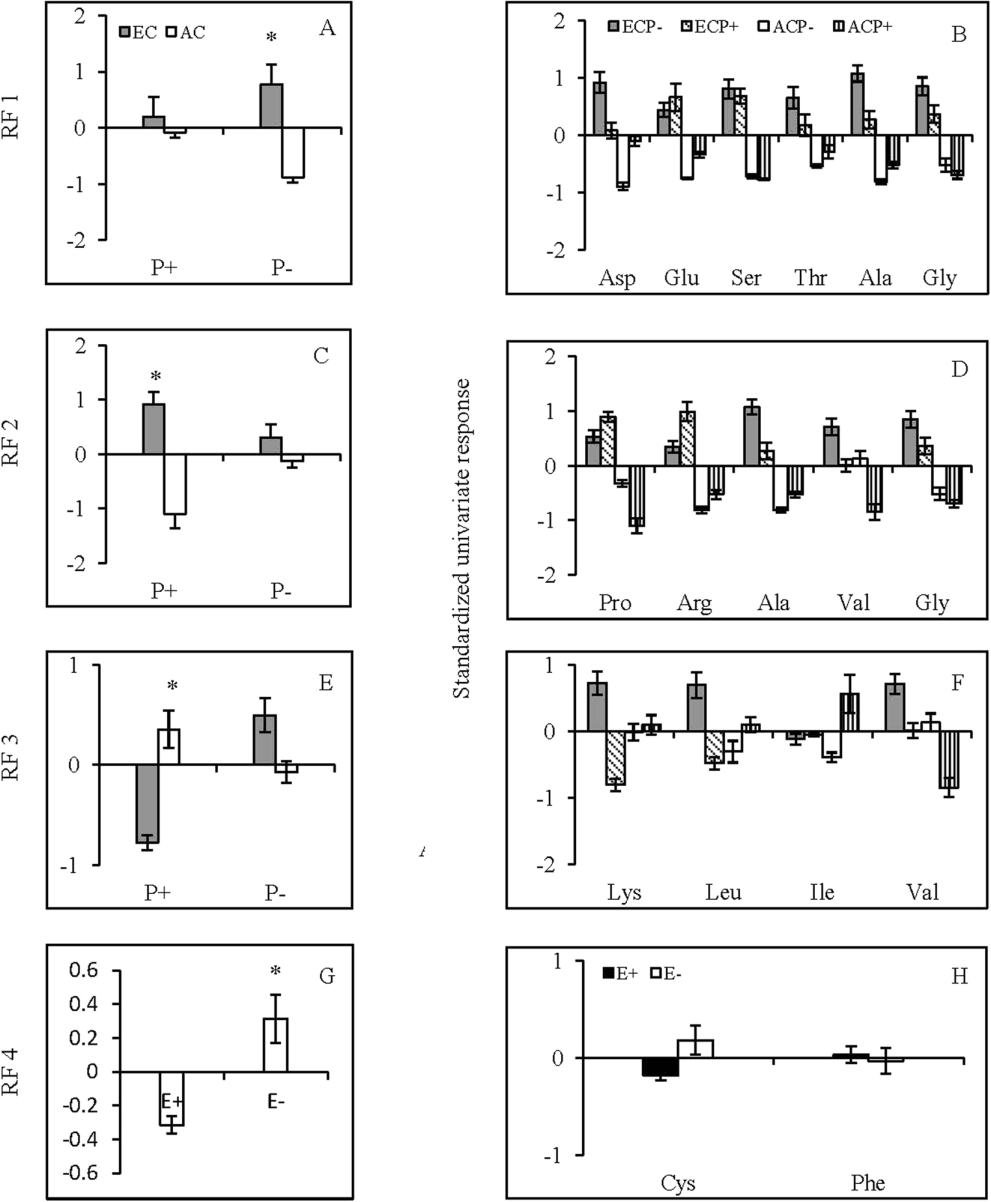
Mean response of rotated factors (RF1–4, **A**,**C**,**E**,**G**) and the standardized univariate response (**B**,**D**,**F**,**H**) of individual amino acids in *Festuca arundinacea* under different endophyte status (E+, endophyte-infected; E−, uninfected), CO_2_ concentration (EC, 800 ppm; AC, 400 ppm), and pathogen inoculation (P+, inoculated by *Curvularia lunata*; P−, uninoculated control). *Meant significant difference at 0.05 level.

Because the responses of the 17 amino acids that were measured were not independent, after measurement, we used a PCA to reduce the number of amino acid response variables to a new set of composite variables. To facilitate interpretation of the principal components, we subjected the first four principal components to factor rotation and retained four rotated factors (RF1, RF2, RF3, and RF4, which accounted for 82.61% of the total variance) (Fig. 6). As the values of the rotated factor increased, the variables that load heavily and positively (loading ≥ +0.5) also increased, while the variables that load heavily but negatively (loading ≤ −0.5) decreased. The standardized univariate responses of these variables are shown in Fig. 7 to facilitate the interpretation of the multivariate responses and to allow a closer inspection of the variables loading heavily onto RF1, RF2, RF3, and RF4.

Six amino acids, Asp, Glu, Ser, Thr, Ala and Gly loaded heavily and positively onto RF1; five amino acids, Pro, Arg, Ala, Val and Gly loaded positively onto RF2; four amino acids, Lys, Leu, Ile and Val loaded positively onto RF3 (Fig. 6). The interaction between elevated CO_2_ and pathogen inoculation significantly affected RF1, RF2 and RF3 (Table 2). In P- group, elevated CO_2_ tended to enhance RF1, RF2 and RF3, but significant effect only occurred on RF1. In P+ group, elevated CO_2_ increased RF2, decreased RF3 but had no effect on RF1 (Fig. 7). Cys loaded heavily and positively onto RF4, and Phe loaded heavily but negatively onto RF4 (Fig. 6). Endophyte infection significantly decreased RF4 (Table 2, Fig. 7).

### Lignin accumulation

**Figure 8:**
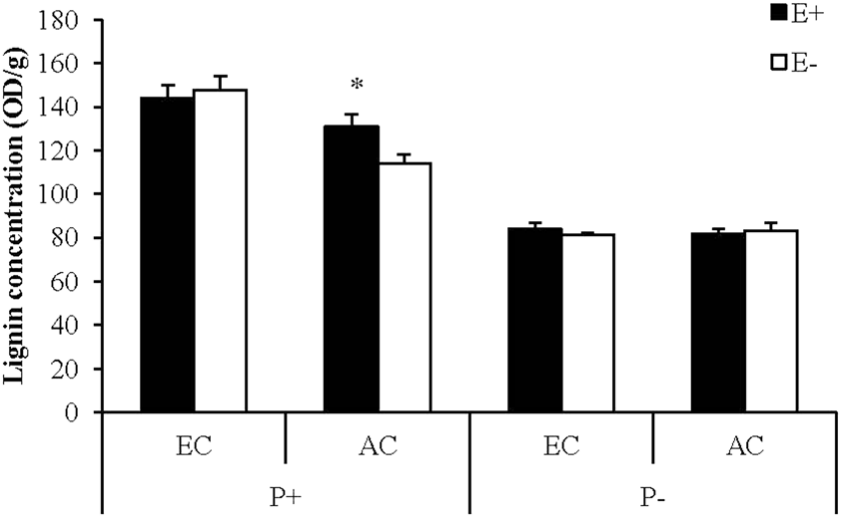
Lignin concentration of endophyte-infected (E+) or uninfected (E−) *Festuca arundinacea* under different CO_2_ concentration (EC, 800 ppm; AC, 400 ppm), and pathogen inoculation (P+, inoculated by *Curvularia lunata*; P−, uninoculated control). *Meant significant difference at 0.05 level.

Lignin concentration was significantly affected by interaction between CO_2_ concentration and pathogen inoculation (Table 2). Pathogen inoculation resulted in lignin accumulation in the leaf of tall fescue under ambient CO_2_ concentration, and this trend was further strengthened by elevated CO_2_ concentration (Fig. 8). Lignin concentration was significantly affected by interactions among CO_2_ concentration, endophyte infection and pathogen inoculation. Only under ambient CO_2_ and pathogen inoculation condition, lignin concentration of the leaf was greater in E+ than in E− plants (Fig. 8).

## Discussion

### Plant growth response

The effects of elevated CO_2_ on growth of plants, especially C_3_ plants, have been widely studied, but most published papers on plant response to elevated CO_2_ fail to even state the endophyte status of their plant material. In the pioneering study, Marks and Clay^29^ found no significant interactions between CO_2_ enrichment and endophyte infection on the growth of perennial ryegrass. Similar results have been reported by Newman, *et al*.^9^ in tall fescue. In contrast, in the present study, we found a significant endophyte infection × CO_2_ interaction for tiller number, maximum net photosynthetic rate and shoot biomass. We found that growth advantage of E+ over E− plants occurred only under high N and ambient CO_2_ conditions. Under high N conditions, elevated CO_2_ improved shoot growth of both E+ and E− plants, but the growth advantage of E+ disappeared under elevated CO_2_. That is to say, elevated CO_2_ counteracted the beneficial effect of endophyte infection on the growth of the host. Although significant endophyte infection × N supply × CO_2_ interaction for growth response has not been reported, this result is consistent with most published reports in that growth advantage of E+ plants occurred under high N conditions^9, 23, 30, 49^, and consist with the results in tall fescue that no significant difference appeared in growth between E+ and E− plants under elevated CO_2_^9, 50^.

In tall fescue, Brosi, *et al*.^51^ found that endophyte infection frequency was significantly higher under elevated CO_2_ compared to ambient; and Ryan, *et al*.^30^ found that endophyte concentration increased under elevated CO_2_. If fungal concentration was correlated with vegetative vigor of the host plant directly^28^, elevated CO_2_ may promote the plant-fungal endophyte mutualism. In the present study, we did find elevated CO_2_ improve the growth of E+ plants, but elevated CO_2_ improve the growth of E− plants in a higher degree, and thus a significant growth difference between E+ and E− plants did not exist anymore under elevated CO_2_. This phenomenon might be related to photosynthetic ability of tall fescue. Tall fescue belongs to a C_3_ grass. Because of the lack of CO_2_ concentrating ability, at ambient CO_2_, its carboxylation function of Rubisco is thought to be limited by CO_2_. With CO_2_ concentration in the air increasing, its photosynthetic rate will increase^3, 52, 53^. Under ambient CO_2_, photosynthetic ability of grasses can be improved by endophyte infection^9, 54–56^. Under elevated CO_2_, the carboxylation function of Rubisco in tall fescue might be near saturation, and the effect of endophyte infection on photosynthesis might be negligible. That is to say, elevated CO_2_ might counteract the beneficial effect of endophyte infection in photosynthesis and thus biomass to the host plants.

### C and N metabolism

Independent of endophyte infection, elevated CO_2_ altered tall fescue tissue chemistry in some expected ways^3, 57–59^, such as increasing carbohydrates (here soluble sugar concentration), decreasing N concentration and thus increasing C:N ratio. As for amino acid concentrations, studies have reported both positive^60, 61^ and negative^30, 62^ effects of elevated CO_2_ on amino acids. In the present study, elevated CO_2_ tended to enhance the concentration of 12 out of 17 amino acids tested. From an herbivore perspective, increased concentrations of soluble sugar and amino acids would increase palatability^63, 64^. However, the subsequent decrease in the percentage of N and the increase in C:N ratio under elevated CO_2_ could offset this impact^62, 65^.

Endophyte infection has been described to result in a reduction of nitrogenous compounds in tall fescue^66, 67^ and ryegrass^8^. In the present study, we found that endophyte infection significantly decreased the soluble sugar concentration, leaf N and increased C:N ratio of the host grass. Endophyte infection had no effect on most amino acids tested, except decreased RF4. Although we found no interaction between CO_2_ and endophyte on soluble sugar, amino acids concentration and C:N ratio, similar to the results from Ryan, *et al*.^30^, we did find significant interaction between CO_2_ and endophyte infection on leaf N concentration. Under ambient CO_2_ and high N conditions, E+ plants had smaller leaf N concentration than E− plants. With CO_2_ elevated, however, no difference between E+ and E− plants occurred. Here, both elevated CO_2_ and endophyte infection can decrease leaf N concentration, but the decreasing degree resulted from CO_2_ was even larger. Under high N conditions, elevated CO_2_ resulted in 54.5% less while endophyte infection resulted in 20.8% less in leaf N concentration. Alkaloids are considered to contribute to defense. Although we did not measure alkaloids in the present study, both Ryan, *et al*.^30^ and Brosi, *et al*.^51^ in tall fescue found that alkaloid production decreased with CO_2_ concentration elevated. Ryan, *et al*.^30^ further suggested that plants where the C:N ratio was highest would have the lowest alkaloid per unit endophyte concentrations. All these results suggest that CO_2_ enrichment might buffer the effect of endophyte infection on the N-metabolism of host plants.

### Pathogen resistance

In terms of the disease resistance of endophyte infection on the host, the beneficial effects of endophyte infection have been reported in perennial ryegrass, tall fescue and native grasses^36, 39, 68^. Pańka, *et al*.^37^ observed stronger susceptibility of E− tall fescue to *Rhizoctonia zeae* than E+ counterparts. A significant increase in resistance to dollar spot disease, caused by *Sclerotinia homoeocarpa*, has also been observed in *Festuca rubra* ^42^. In the present study, we found that endophyte infection improved pathogen resistance of tall fescue, but the significant effect occurred only under ambient CO_2_ concentration.

In studies examining plant response to fungal disease under elevated CO_2_, disease incidence and severity are variable, from decreased^69, 70^, unchanged^71, 72^ to increased^73^. When endophyte infection was considered, up to now, we found no report on its contribution to the host grasses under elevated CO_2_. In the present study, we found that disease severity of both E+ and E− plants decreased under elevated CO_2_.

The interesting result in the present study is that the advantage of E+ over E− plants in pathogen resistance under ambient CO_2_ disappeared with CO_2_ elevated. One possible explanation might be that the nutritive quality of leaves is responsible for pathogen development. Thompson and Drake^74^ found positive correlations existed between plant N concentration and disease severity. And this correlation has been proved by Mcelrone, *et al*.^75^ and Plessl, *et al*.^70^. In the present study, the main effects of elevated CO_2_ and endophyte infection were similar on reducing leaf N concentration and decreasing pathogen severity, but the contribution of elevated CO_2_ was even bigger. So it might be larger degree reduction of N concentration resulted from elevated CO_2_ that cover up the role of endophyte infection on N concentration and thus pathogen resistance. In the present study, we further found that both elevated CO_2_ and endophyte infection resulted in lignin accumulation in tall fescue after pathogen inoculation. Lignin is one of important phenolic compounds, whose deposition is believed to play a crucial role in barricading the pathogen from invading the plant through physical exclusion^76^. In the present study, lignin accumulation went along with a decrease in susceptibility and might be a factor contributing to pathogen resistance^77, 78^.

Our results shed some light of the effects of elevated CO_2_ on the mutualistic relationship between a grass and a fungus. Besides CO_2_ concentrations, other factors such as temperature and water availability are likely to be altered in coming years^1^. Therefore, the response of grass-endophyte symbiosis to pathogens will be more complex and depend largely on the specific environmental conditions encountered. Given the extensive acreage of tall fescue worldwide and the fact that the ecological effects of this grass–fungal endophyte symbiosis have been observed at population, community, and ecosystem-scales^79^, understanding the response of tall fescue and its endophytic fungi to climate change may be important in predicting not only the responses of pathogens, but also grazing herbivores and ecological processes such as litter decomposition and nutrient cycling.

## Conclusions

Our experiments provided evidence that endophyte infection improved the growth of tall fescue, but this benefit was affected by elevated CO_2_ and N supply. Only under ambient CO_2_ and high N conditions, both maximum net photosynthetic rate and shoot biomass were greater in E+ than in E− plants. With CO_2_ concentration elevated, the beneficial effect of endophyte infection on the growth disappeared. Similarly, endophyte infection can enhance resistance of tall fescue towards *Curvularia lunata* only under ambient CO_2_. Elevated CO_2_ counteracted the beneficial effect of endophyte infection on the growth and pathogen resistance of the host grass.

## Materials and Methods

### Plant material

Endophyte-infected (E+) seeds of tall fescue (*Lolium arundinaceum* Darbyshire ex. Schreb., KY-31) were naturally infected with *Epichloë coenophialum* ^80, 81^, and uninfected (E−) seeds were acquired by eliminating the endophyte through the long-term storage of E+ seeds at room temperature. This procedure reduces the viability of the endophyte but not the seeds^82^. E+ and E− seeds were originally obtained from Professor Keith Clay at Indiana University, USA. The seeds used in this experiment were several generations distant from the storage treatment and came from freely cross-pollinated field-grown parents. To re-isolate the endophyte, 30 E+ and 30 E− plant individuals were randomly sampled, and the method described by Latch & Christensen^83^ was used with a slight modification that the time for sodium hypochlorite treatment was 8–10 min, and the petri plates containing potato dextrose agar (PDA) were incubated in the dark at 25 °C. Up to 4 weeks’ examination, only one species of endophyte, *E*. *coenophialum*, was isolated from E+ seedlings while no endophyte was found in E− seedlings. Meantime, seed germination rates for E+ and E− seeds were compared before the experiment. No significant differences were found between them, with regard to the number of days to first seedling emergence and germinations rates. Four weeks later, seven equally sized seedlings were transferred into each plastic pot (15 cm × 13.5 cm) filled with 1.4 kg of sterilized sand. After a week’s growth, they were differently treated and were placed into two separate growth chambers set at 400 or 800 ppm CO_2_. Plants were maintained at 30000 lux and a 12/12 h light/dark cycle at 25/20 °C, respectively. Endophyte status of the plants was checked both immediately before and after the experiment by microscopic examination from leaf sheaths stained with aniline blue described by Latch & Christensen^83^. We found that seedlings from E+ seeds were all infected (100%) while no seedling from E− seeds was infected (0%).

### Experiment design

The present study included two experiments. In the first experiment, we addressed the questions: does endophyte improve growth of the grass host under elevated CO_2_ concentration? If this is the case, how does nitrogen (N) availability affect the symbiosis-dependent benefits? In the second experiment, we addressed the question: does elevated CO_2_ affect pathogen resistance of grass-endophyte symbiont? From the first experiment, we found that endophyte-associated benefit only occurred in high N condition. So in the second experiment, test was performed only in high N level.

### Experiment 1

A three factors randomized block design was used in this experiment. The first factor was two CO_2_ concentrations with two levels: ambient CO_2_ (400 ppm, AC) and elevated CO_2_ (800 ppm, EC). The second factor was N availability with two levels: high N (HN) and low N (LN). The third factor was endophyte infection status: endophyte-infected (E+) and uninfected (E−). Each treatment was replicated five times, totally 40 pots.

The nutrients were supplied by the addition of a modified Hoagland nutrient solution. The composition of the nutrient solution was 5.0 mM CaCl_2_, 5.0 mM KCl, 2.5 mM MgSO_4_·7H_2_O, 2.0 mM KH_2_PO_4_, 29 μM Na_2_-EDTA, 20 μM FeSO_4_·7H_2_O, 45 μM H_3_BO_3_, 6.6 μM MnSO_4_, 0.8 μM ZnSO_4_·7H_2_O, 0.6 μM H_2_MoO_4_, 0.4 μM CuSO_4_·5H_2_O, and pH 6.0 ± 0.1. Nitrogen was added in the form of NH_4_NO_3_, which was delivered as 1 mM N (LN) or 10 mM N (HN), respectively. During the experiment, 100 ml of nutrient solution was added once a week to each pot, a total of 9 times. Plants were watered as necessary with deionized water. In each block, the positions of the pots were randomly rotated each week to minimize location effects. The experiment lasted for 63 days.

### Experiment 2

A three factors randomized block design was used in this experiment. The first factor was two CO_2_ concentrations with two levels: ambient CO_2_ (400 ppm, AC) and elevated CO_2_ (800 ppm, EC). The second factor was pathogen inoculation with two levels: uninoculated control (P−) and inoculated by *Curvularia lunata* (P+). The third factor was endophyte infection status: E+ and E−. Each treatment was replicated five times, totally 40 pots. Pathogen inoculation was performed after 8 weeks’ growing in the growth chamber with different CO_2_ concentrations. All treatments were sampled at the 6th day after pathogen inoculation.

### Response variables in Experiment 1

#### Photosynthesis parameters

At the end of experiment 1, gas exchange measurements were made on the youngest fully expanded attached leaf in a pot with a LI-COR 6400 infrared gas analyzer (LI-Cor, Lincoln, NE, USA). Under 400 μmol mol^−1^ or 800 μmol mol^−1^ CO_2_, net photosynthetic rate (Pn) was measured at 1,500, 1,200, 1,000, 800, 600, 400, 200, 150, 100, 50, 20 and 0 μmolm^−2^s^−1^ PPFD (photosynthetic photon flux density). According to Pn-PPFD curve, Pmax were determined.

#### Growth and biomass

At the end of experiment 1, regular measurement of tiller number, leaf number, and shoot height of the longest tiller were made on all ramets. Then, the shoot and the root were harvested separately. The harvested material was ven-dried at 80 °C for biomass measurement and C and N analyses.

#### Carbon (C) and nitrogen (N) concentration

C and N concentrations were determined using the dry combustion method with an Elemental Analyser (Vario EL/micro cube, Elementar, Hanau, Germany).

### Response variables in Experiment 2

#### Pathogen inoculation and lesion index recorded

*C*. *lunata* was obtained from Grassland Protection Institute, Lanzhou University, China. It was originally isolated from *Poa pratensis*. For inoculum, the pathogen was cultured on PDA at 25 °C for 2 weeks. Spores were washed with sterile distilled water and filtered through two-layer sterile gauze. A haemocytometer was used to count the spores, and the spore concentration was 13.44 × 10^5^/ml. Plants were inoculated by spraying the spore suspensions using a sprayer until small droplets were seen on the leaves^84^, and the control was sprayed with sterile distilled water. After inoculation, plants were immediately covered with a plastic bag for 36 h to maintain humidity.

Ten fully expanded mature leaves per pot were chosen for measuring the number and length of disease lesions. After measurement, pathogen spore concentration on the leaves was decided according to Nan & Li^84^.

#### Soluble sugar, amino acid and lignin

Soluble sugar content was analyzed using the phenol-sulphuric acid method according to Buysse and Merckx^85^. Amino acids were analyzed by reverse-phase high-performance liquid chromatography (HPLC, Waters 1500-series) with pre-column derivatization using dinitroflurobenzene (DNBF) according to Li and Sun^86^. Lignin measurement was according to the procedure of Reddy, *et al*.^87^.

#### Statistical analyses

For the amino acids, we performed a principal components analysis (PCA) on the correlations among the 17 response variables and then performed factor rotation using the varimax method^63, 88^. After varimax rotation, we retained four rotated factors (RF). The RF variables and all other indexes were subjected to three-way analyses of variance (ANOVA). Differences between the means were compared using Duncan’s multiple-range tests at P < 0.05. All statistical analyses were performed using SPSS 21.0 software.
